# Serum levels of N–terminal proB–type natriuretic peptide in mechanically ventilated critically ill patients – relation to tidal volume size and development of acute respiratory distress syndrome

**DOI:** 10.1186/1471-2466-13-42

**Published:** 2013-07-09

**Authors:** Rogier M Determann, Annick ANM Royakkers, Jacqueline Schaefers, Anita M de Boer, Jan M Binnekade, Jan P van Straalen, Marcus J Schultz

**Affiliations:** 1 Department of Intensive Care Medicine, Academic Medical Centre, University of Amsterdam, Meibergdreef 9, Amsterdam 1105 AZ, The Netherlands; 2 Laboratory of Experimental Intensive Care and Anesthesiology (L·E·I·C·A), Academic Medical Centre, University of Amsterdam, Amsterdam, The Netherlands; 3 Department of Internal Medicine, Academic Medical Centre, University of Amsterdam, Amsterdam, The Netherlands; 4 Laboratory of Clinical Chemistry (LAKC), Academic Medical Centre, University of Amsterdam, Amsterdam, The Netherlands; 5 Department of Anesthesiology, Tergooi Hospitals, Blaricum, The Netherlands

**Keywords:** Mechanical ventilation, Acute lung injury, Tidal volume, Ventilator–associated lung injury, NT–proBNP

## Abstract

**Background:**

Serum levels of N–terminal proB–type natriuretic peptide (NT–proBNP) are elevated in patients acute respiratory distress syndrome (ARDS). Recent studies showed a lower incidence of acute cor pulmonale in ARDS patients ventilated with lower tidal volumes. Consequently, serum levels of NT–proBNP may be lower in these patients.

We investigated the relation between serum levels of NT–proBNP and tidal volumes in critically ill patients without ARDS at the onset of mechanical ventilation.

**Methods:**

Secondary analysis of a randomized controlled trial of lower versus conventional tidal volumes in patients without ARDS. NT–pro BNP were measured in stored serum samples. Serial serum levels of NT–pro BNP were analyzed controlling for acute kidney injury, cumulative fluid balance and presence of brain injury. The primary outcome was the effect of tidal volume size on serum levels of NT–proBNP. Secondary outcome was the association with development of ARDS.

**Results:**

Samples from 150 patients were analyzed. No relation was found between serum levels of NT–pro BNP and tidal volume size. However, NT-proBNP levels were increasing in patients who developed ARDS. In addition, higher levels were observed in patients with acute kidney injury, and in patients with a more positive cumulative fluid balance.

**Conclusion:**

Serum levels of NT–proBNP are independent of tidal volume size, but are increasing in patients who develop ARDS.

## Background

Both atelectasis and fibrosis may decrease pulmonary compliance in patients with acute respiratory distress syndrome (ARDS) [1]. Together with hypoxic pulmonary vasoconstriction they may increase pulmonary vascular resistance and cause pulmonary hypertension. ARDS may thus lead to an acute cor pulmonale [2]. Right ventricular dysfunction occurs in 61% of patients with ARDS submitted to mechanical ventilation using conventional tidal volumes [3]. It is estimated to be present in only 25% of patients with ARDS submitted to mechanical ventilation using lower tidal volumes [4].

N–terminal proB–type natriuretic peptide (NT–proBNP) is secreted by the ventricles of the heart in response to excessive stretching of heart muscle cells [5]. Although the right ventricle secretes less NT–proBNP than the left ventricle [5,6] serum levels of NT–proBNP can be elevated in patients with an acute cor pulmonale due to ARDS [7,8]. Notably, stretching of heart muscle cells could also be the result of fluid overload, frequently seen in patients with ARDS. In addition, NT–proBNP levels can be elevated due to kidney failure [9] and elevated levels are seen in patients with acute brain injury [10].

It can be questioned whether the size of tidal volumes used with mechanical ventilation influences the right ventricular afterload and therefore has an effect on serum levels of NT–proBNP. In the present study we measured levels of NT–proBNP in serial samples taken from patients without ARDS who were randomized to mechanical ventilation with lower tidal volumes or conventional tidal volumes in a previous trial [11]. We hypothesized that conventional tidal volumes may lead to increased pulmonary vascular resistance as the incidence or ARDS is higher compared to lower tidal volumes [11]. In this way, increased pulmonary vascular resistance may lead to increased stretching of the right ventricle and therefore increased NT-proBNP levels. To investigate this possibility we studied the relation between tidal volumes and NT–proBNP levels. The purpose of this study was (a) to investigate whether serum levels of NT–proBNP are dependent on tidal volume size in patients without ARDS, and (b) serum levels of NT–proBNP to increase parallel to severity of lung injury, when controlling for acute kidney injury, cumulative fluid balance and presence of brain injury.

## Methods

The original trial was approved by the medical ethics committee of the Academic Medical Center, Amsterdam, The Netherlands, and registered at the Netherlands Trial Register (http://www.trialregister.nl; NTR151) [11]. Informed consent was acquired from patient or their closest representative prior to inclusion. Measurement of levels of NT–proBNP in stored samples was preplanned; there was no need to ask for additional informed consent.

### Patients

Patients without ARDS and an anticipated duration of mechanical ventilation of more than 2 days were eligible for the trial as described previously [11]. Randomization, data and sample collection had to be started within 36 hours of the start of mechanical ventilation. Exclusion criteria were: age under 18 years, participation in other clinical trials, pregnancy, history of severe chronic obstructive pulmonary disease or severe restrictive pulmonary disease, history of pneumectomy or lobectomy, and pulmonary thromboembolism. In the original trial patients were randomized to either mechanical ventilation with conventional tidal volumes (10 ml/kg) or lower tidal volumes (6 ml/kg) until they were weaned from the ventilator.

### Diagnosis of acute lung injury

During the trial patients were assessed for development of lung injury. All patients meeting the North–American European Consensus Conference criteria for ARDS were classified as having lung injury [12]. For this two independent observers were asked to review all patient charts. In case of disagreement, consensus had to be obtained while reviewing the patient together.

### Diagnosis of acute kidney injury, brain injury and heart failure

Presence of acute kidney injury (AKI) was scored with use of the RIFLE criteria [13]. Patients with intracranial hemorrhage (epidural, subdural, subarachnoidal or intraparenchymal hemorrhage) and with brain infarction were classified as having brain injury. Patients with compromised cardiac function, as seen with echocardiography, were classified as having heart failure. Echocardiography was performed routinely in patients with myocardial infarction and in patients with suspected cardiac dysfunction. Cardiac dysfunction was suspected in case of hemodynamic instability which could not be explained by bleeding or an event causing vasodilatation such as sepsis, allergic reaction, or administration of agents causing vasodilatation. Patients were classified as having heart failure if the left ventricle ejection fraction was reduced below 50% or if diastolic dysfunction was present classified as an E/A ratio < 1.

### Data collection

At inclusion, demographic data, ventilation parameters and clinical and radiological data were recorded. Each second day, ventilator settings, blood gas parameters, and radiographic data were recorded until the patient was weaned from the ventilator. In addition, lung compliance, oxygenation index [14] and lung injury score (LIS) were calculated [15].

On the day of enrolment and each second day until the patient was weaned from the ventilator the cumulative fluid balance was calculated, taking into account all types and infused volumes of fluids (including blood products, colloids, saline solutions), (parenteral and/or enteral) feeding, urine output and other fluid losses. On the day of enrollment the fluid balance was calculated from day of ICU–admission.

### Sample collection

On the day of enrolment and each second day until the patient was weaned from the ventilator blood samples were drawn from an indwelling arterial catheter.

### Measurements

Serum levels of NT–proBNP were measured using a commercially available electrochemiluminescence immunoassay (Roche Diagnostics Nederland BV, Almere, the Netherlands).

### Statistical analysis

The association between NT-proBNP levels and tidal volume was the primary endpoint of the study and the association with acute respiratory distress syndrome was a secondary endpoint. Tidal volumes were expressed as ml per kilogram ideal body weight. Ideal body weight was calculated as described before [11]. Data are presented as mean (with standard deviation) for parametric data or medians (with interquartile range) for non–parametric data. Baseline data were compared using the student’s t–test, Mann–Whitney U–test, Chi–square test or Fisher exact test where appropriate. A multivariate regression model was constructed to study NT–proBNP levels controlling for tidal volume size, ARDS, cumulative fluid balance, and presence of AKI. For calculations the original data were log transformed if the distribution was not normal. Statistical analyses were performed using Statistical Package for the Social Sciences 17.0 (SPSS Inc, Chicago, Illinois, USA). A *P*–value of < 0.05 was considered statistically significant.

## Results

### Patients

A total of 150 patients were included in the original randomized controlled trial and all patients were part of the present study. Of these, 99 patients (66%) were male. They were 61 ± 16 years old. Baseline characteristics and admission diagnoses are presented in Table 1. At baseline 57 patients (38%) were diagnosed with heart failure, 50 patients (33%) had brain injury, and 63 patients (42%) classified as having AKI. There were significantly more patients with heart failure in the lower tidal volume group. The cumulative fluid balance at study entry was comparable between groups. During the study 12 patients (8%) developed ARDS.

**Table 1:** Demographic data

	**V**_**T**_**10 ml/kg (n = 74)**	**V**_**T**_**6 ml/kg (n = 76)**	***P*–value**
Age (years)	58 (± 17)	63 (± 15)	0.06
Male sex	50 (68%)	49 (64%)	0.69
APACHE II–score	20 (± 8)	21 (± 7)	0.93
SOFA score	8 (± 4)	7 (± 3)	0.19
LIS	1.2 (± 0.6)	1.3 (± 0.6)	0.08
P/F	40.0 (± 18.9)	36.0 (± 11.4)	0.14
Fluid balance before randomization (liters)	2.2 (± 2.7)	1.8 (± 1.7)	0.28
Presence of brain injury	29 (39%)	21 (28%)	0.13
Presence of heart failure	22 (30%)	35 (46%)	0.04
Presence of kidney injury	31 (42%)	32 (42%)	0.97
Risk	17 (23%)	14 (18%)	
Injury	8 (11%)	13 (17%)	
Failure	6 (8%)	5 (7%)	

*APACHE*–II acute physiology and chronic health evaluation–II, *SOFA* sequential organ failure assessment, *LIS* lung injury score. PF PaO_2_ to FiO_2_ ratio.

### Baseline serum levels of NT–proBNP

Serum levels of NT–proBNP at baseline were 1640 [494–4743] pg/ml. In patients with heart failure levels were 1980 [830–4200] pg/ml versus 960 [360–5050] pg/ml in patients without heart failure (*P* = 0.32). In patients with AKI levels were 3600 [760–11460] pg/ml versus 920 [370–3000] pg/ml in patients without AKI (*P* < 0.001). In patients with brain injury levels were 510 [200–2570] pg/ml versus 2710 [870–6140] pg/ml in patients without brain injury (*P* < 0.001).

### Serial serum levels of NT–proBNP

**Figure 1:**
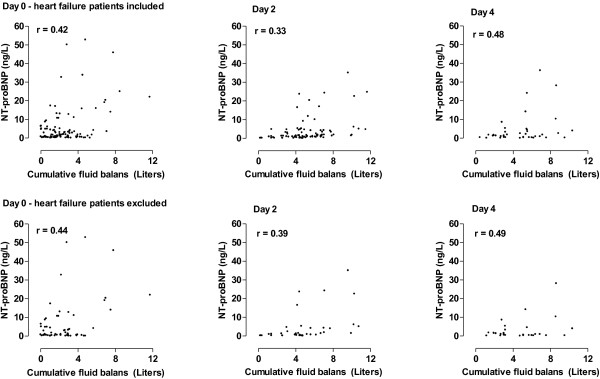
**Upper panels: serum levels of NT–proBNP in a scatterplot with cumulative fluid balance on day 0 to 4; all correlations (r) were significant on all days.** Lower panels: serum levels of NT–proBNP in patients without heart failure. All correlations were significant on all days.

**Figure 2:**
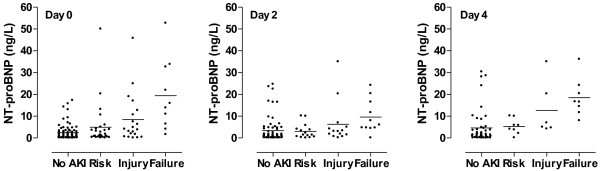
**Serum levels of NT–proBNP in patients grouped in RIFLE grades of acute kidney injury on day 0 to 4.** Patients with failure had significantly higher levels as compared to the other groups on all days.

Serum levels of NT–proBNP strongly correlated with cumulative fluid balances (*P* < 0.001, Figure 1). This was true for all patients at all time points, including patients who were classified as not having heart failure. Serum levels of NT–proBNP also strongly correlated with the development of AKI. Patients with AKI had significantly higher serum levels of NT–proBNP as compared to patients who were at risk or had injury at all time points (*P* < 0.001; Figure 2).

### Serum levels of NT–proBNP and tidal volume size and development of ARDS

**Figure 3:**
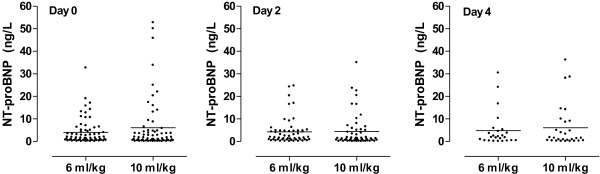
Serum levels of NT–proBNP on day 0 to 4 in patients ventilated with tidal volumes of 6 ml/kg versus 10 ml/kg.

**Figure 4:**
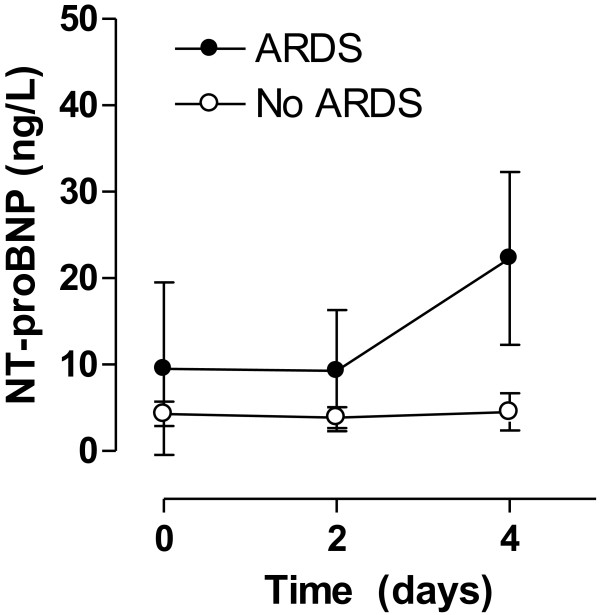
**Serum levels of NT–proBNP in patients without acute respiratory distress syndrome (ARDS) and patients developing ARDS.** Serum levels of NT–proBNP were significantly higher in patients with ARDS on day 4.

There was no association between tidal volume size and serum levels of NT–proBNP at all time points (Figure 3). This was also true in case all patients with heart failure were excluded from the analysis. Tidal volume was not associated with hypercapnia at all time points (t = 0 days, *P* = 0.69; t = 2 days, *P* = 0.51; t = 4 days, *P* = 0.24). The serum NT–proBNP levels were not associated with the partial carbon dioxide level at all time points (t = 0 days, *P* = 0.11; t = 2 days, *P* = 0.50; t = 4 days, *P* = 0.71). The serum NT–proBNP levels increased in patients who developed ARDS, while levels remained unchanged in patients who did not develop ARDS (Figure 4, *P* = 0.03).

### Multivariate analysis

Multivariate analysis showed development of ARDS (*P* = 0.005), cumulative fluid balance (*P* = 0.006), and presence of kidney injury (*P* = 0.007) all to be independently associated with serum levels of NT–proBNP (linear mixed models). Multicollinearity was not a major problem as covariances were low (<0.03) for all pairs of variables.

## Discussion

The results of this study can be summarized as follows: (1) serum levels of NT–proBNP are not influenced by tidal volume size in patients without ARDS at onset of mechanical ventilation; (2) serum levels of NT–proBNP are dependent on the development of ARDS; and (3) serum levels of NT–proBNP are dependent on cumulative fluid balances, both in patients with and without HF, and on development of AKI.

Several limitations of our study have to be acknowledged. First, we may not have included a sufficient number of patients to demonstrate a difference in serum levels of NT–proBNP between the randomization groups. Notably, as patients developing ARDS in the conventional tidal volume group were immediately set to lower tidal volume ventilation as soon as ARDS was recognized, we may have limited right ventricular wall stress, possibly influencing serum levels of NT–proBNP. Second, we did not perform pulmonary artery pressure measurements to assess the afterload of the right ventricle. However, this procedure can result in complications as cardiac arrhythmias or lung bleeding. We therefore choose not to perform this procedure in patients who were already compromised by ARDS. Third, our study was performed using the American-European consensus conference ARDS criteria. The ARDS criteria have recently been changed [16]. In our study patients who developed ARDS all had underlying risk factors [11]. Furthermore, it is standard practice in our clinic to perform echocardiography in case heart failure is suspected. As all patients were ventilated with at least 5 cm H2O of PEEP, ARDS diagnosis may not have differed significantly in case patients were reviewed based on the Berlin definition.

The incidence of acute cor pulmonale with ARDS seems to have declined with the use of lower tidal volumes. The study by Vieillard-Baron *et al*. showed a lower incidence of acute cor pulmonale diagnosed by echocardiography [4]. They suggested that lowering tidal volumes with mechanical ventilation resulted in afterload reduction of the right ventricle, thereby minimizing the incidence of acute cor pulmonale. Their results also suggested that a lower tidal volume may be associated with higher degree of hypercapnia which was significantly associated with presence of acute cor pulmonale. In our study no correlation was found between tidal volume size and serum levels of NT–proBNP. We also did not find any relation between hypercapnia and the level of NT–proBNP. This may have been caused by the two independent determinants of NT–proBNP levels. i.e., the cumulative fluid balance and the presence of kidney injury. Although ARDS is associated with hypercapnia, patients with higher cumulative fluid balance or with AKI were not hypercapnic in most cases.

The relation between tidal volume size and acute cor pulmonale may be different in patients with and without ARDS. Although higher tidal volumes may indeed increase the afterload of the right ventricle, this may be less or even not important in patients without lung disease. However, the effect of conventional tidal volume on the right ventricle and therefore on serum levels of NT–proBNP may have been tempered in our study. In patients who developed ARDS, the tidal volume was immediately set at 6 ml/kg in patients of the conventional tidal volume group. As mentioned above, this may have resulted in less stress on the right ventricle and accordingly lower serum levels of NT–proBNP.

In line with previous studies, serum levels of NT–proBNP correlated with cumulative fluid balance [17,18] and the presence of AKI [19]. Our finding that serum levels of NT–proBNP were associated with both cumulative fluid balance, and AKI and ARDS may disqualify this protein as a suitable biological marker to distinguish between ARDS and acute pulmonary cardiogenic edema or acute pulmonary edema due to circulatory overload. Recent studies have investigated this issue. Rana *et al*. studied patients with acute pulmonary edema and measured serum levels of NT–proBNP within 24 hours after the onset of the edema [20]. They found a low serum level of NT–proBNP to be supportive of ARDS. However levels of NT–proBNP were not discriminative in patients with a level above 250 pg/ml. In a study of patients with acute hypoxic respiratory failure and bilateral pulmonary infiltrates, low serum levels of NT–proBNP (< 200 pg/ml) were found to be supportive of ARDS while high serum levels of NT–proBNP (> 1200 mg/ml) were supportive of cardiogenic pulmonary edema [21]. However, in a study of patients with respiratory distress and bilateral pulmonary edema, Levitt *et al*. could not find a useful cut–off point for NT–proBNP in diagnosing ARDS [22]. Finally, in a study of patients with respiratory distress after blood transfusions, Li *et al*. observed higher serum levels of NT–proBNP in patients with circulatory overload compared to patients with transfusion–associated lung injury, but NT–proBNP had no diagnostic value due to a large range of overlapping values [17]. All studies bear more of less the same message: in patients with acute respiratory distress, serum levels of NT–proBNP tend to be lower in patients with ARDS as compared to patients with circulatory overload or congestive heart failure.

## Conclusions

Serum levels of NT–proBNP are independent of tidal volume size in patients without ARDS at the onset of mechanical ventilation. Presence of ARDS, a more positive cumulative fluid balance and AKI are all independently associated with increased serum levels of NT–proBNP. NT–proBNP cannot to be used as biological marker of ventilator–induced lung injury.

## Competing interests

The authors declare that they have no competing interests.

## Authors’ contributions

RD participated in the design and coordination of the study, performed the statistical analyses, and drafted the manuscript. AR participated in the design and coordination of the study. JS acquired the clinical data, and drafted the manuscript. AB acquired the clinical data and helped in the performing the immunoassays. JB performed the statistical analyses. JS performed the immunoassays. MS participated in the design and coordination of the study. All the authors read and approved the final version of the manuscript.

## Pre-publication history

The pre-publication history for this paper can be accessed here:

http://www.biomedcentral.com/1471-2466/13/42/prepub
